# Interferon Inhibition in SLE: From Bench to Bedside

**DOI:** 10.31138/mjr.010324.iis

**Published:** 2024-06-30

**Authors:** Dimitrios Deligeorgakis, Elpida Skouvaklidou, Christina Adamichou

**Affiliations:** Department of Rheumatology, 4th Department of Internal Medicine, Hippokration Hospital, Thessaloniki, Greece

**Keywords:** type I interferon, anifrolumab, systemic lupus erythematosus, efficacy, safety

## Abstract

Despite advances in the management of systemic lupus erythematosus (SLE), it remains a chronic disease with frequent flares, requiring constant medical care, laboratory exams, hospitalisations, and the use of immunosuppressive drugs and corticosteroids, increasing the morbidity and mortality of these patients. The past decade of research has brought to light multiple observations on the role of interferons (IFNs) in the pathogenesis of SLE, which paved the way for the development of potential novel therapies targeting the interferon pathway. Following two phase III trials, anifrolumab, a monoclonal antibody which binds to the type I IFN receptor, blocking the activity of type I IFNs, was approved for active SLE. This review summarises the latest research on the role and mechanisms of type I IFNs in SLE and the development and advances on new therapeutic drugs based on IFN inhibition for SLE.

## INTRODUCTION

Systemic lupus erythematosus (SLE) is a complex autoimmune disease, characterised by great clinical heterogeneity as it can affect literally every system and organ with varying degrees of severity. It predominantly affects women of reproductive age, with an estimated female to male ratio of 9:1, while ten to twenty percent of all patients with SLE are diagnosed during childhood, with a lower female/male ratio and approximately 10 percent have disease onset after the age of 50.^1,2^

Despite advances in the diagnosis and management of SLE, it remains a chronic disease with a course characterised by alternating periods of remission and relapse and significant morbidity due to irreversible organ damage as a result of chronic inflammation, corticosteroid use and comorbidities.^3,4^ Therefore, the development of novel therapies that will achieve long periods of remission with reduced corticosteroid use, remain an unmet need. The approval of belimumab, the first biologic therapy for SLE, marked a major advance in the management of the disease, paving the way for the development of further biological agents. Belimumab is an anti-B lymphocyte stimulator monoclonal antibody^5^ and was approved for the treatment of active SLE in 2011, further extended for treatment of active lupus nephritis (LN) as an add-on to standard-of-care (SOC).^6^

More recently, literature has emerged that offers multiple observations on the role of interferons (IFNs), a type of cytokines important for fighting viral infections and regulating the immune system, in the pathogenesis of SLE.^7^ Following two phase III trials, anifrolumab, a monoclonal antibody which binds to type I IFN receptor subunit 1 (IFNAR1), inhibiting the activity of type I IFNs, was approved for active SLE.^8^

In this review, the latest research about the role and mechanisms of type I IFNs in SLE development and advances on new therapeutic drugs based on IFN inhibition for SLE are summarised.

## THE FAMILY OF INTERFERONS

Interferons belong to a family of signalling proteins released by host cells in response to the presence of pathogens, typically during viral and bacterial infections.^9^ Their name derives from their ability to “interfere” with virus replication by protecting cells from viral infections.^10^ However, their function is not limited to fighting pathogens, since they also have antitumor, antiproliferative and immunomodulatory effects.^11^ More specifically, IFNs activate immune cells, such as natural killer (NK) cells and macrophages, upregulate antigen presentation by increasing major histocompatibility complex (MHC) antigen expression, thereby increasing host defence.^9^

Based on their distinct structures, biological activities and the type of receptor through which they signal, human interferons have been classified into three major types: type I (alpha, beta, epsilon, kappa and omega), type II (gamma) and type III (lambda).^9^ Type I IFN alpha (IFN-α) are further divided in 12 different subtypes.^12^ IFNs belonging to all three classes are important both for fighting viral infections and for the regulation of the immune system. Virtually all cell types can express type I and III IFNs, following recognition of viral components, especially nucleic acids, by cytoplasmic and endosomal receptors, although the plasmacytoid dendritic cell (pDC) is considered the natural IFN-producing cell. Emerging evidence shows that cellular sources of IFNs can vary during different viral infections.^13^ Specifically, during respiratory infections, cells lining the airways, like epithelial cells and alveolar macrophages, provide the primary source of type I IFN, during rotavirus infections, epithelial cells of the gastrointestinal system produce type I IFN, while neurons are critical sources of IFN-I during specific brain infections.^13^ In SLE, there is emerging evidence that apart from pDCs, other types of cells, such as keratinocytes, monocytes and neutrophils are involved in IFN production.^14^ On the other hand, type II interferon expression is restricted to immune cells such as T cells and NK cells and is induced by cytokines such as IL-12.^13,15^

## IFN SIGNALLING

Induction of type I IFNs typically occurs when pattern recognition receptors (PRRs) localised in the cytoplasm or in the endosome of cells, sense the presence of viruses, bacteria or microbial nucleic acids.^14,16^ These PRRs include membrane bound Toll-like receptors (TLRs), the cytoplasmic receptors retinoic acid inducible gene 1 (RIG-I)–like receptors (RLRs) and nucleotide oligomerisation domain–like receptors (NLRs).^14,16^ After their release, Type I IFNs all bind to the same ubiquitously expressed type I IFN receptor (IFNAR) that consists of two polypeptide chains of IFNAR1 and IFNAR2. Subsequently, the signalling pathway involves activation of Janus kinase (JAK) 1 and tyrosine kinase (TYK) 2 and formation of the interferon-stimulated gene factor 3-complex (IGSF3), which includes signal transducer and activator of transcription (STAT)1, STAT2 and interferon regulatory factor (IRF). IGSF3 moves into the cell nucleus and binds to specific nucleotide sequences, called IFN-stimulated response elements (ISREs), which induce new gene transcription (IFN-regulated genes) in order to mediate antiviral responses.^12^

The type II IFN comprise of IFN-γ which binds to the IFN-γ receptor (IFNGR) which is expressed on most cells.^15,17^ Ligation of the IFNGR results in phosphorylation of STAT1 homodimers, via activation of JAK1 and JAK2, and binding to IFN-γ-activated sites (GASs) and subsequent gene expression. Induction of type I and II genes is largely overlapped, since the latter’s signalling pathway can also be used by IFNAR.^15,17^

Type III IFNs consist of four newly identified lambda IFNs: IFNλ1/IL29, IFNλ2/IL28A, IFNλ3/IL28B and IFNλ4.^16,18^ IFN-λs are mostly found at barrier surfaces and are produced by epithelial and epithelial-origin cells of the respiratory and gastrointestinal tracts. The type III IFNs signal through a receptor complex (IFNLR1/IL10Rβ) that is primarily expressed on gastrointestinal, respiratory and urogenital epithelial cells, hepatocytes and a few immune cells including neutrophils and DCs.^16,18^

## IFN SIGNATURE

IFNs have been intensively investigated recently due to their crucial role in a number of immunological pathways involved in autoimmune diseases, summarised by the term “IFN signature”. The so-called IFN signature refers to the evidence of an upregulation of transcripts induced by the different IFN subtypes.^17,19^ Until recently, the term did not allow differentiation between the three families of IFNs, whereas in recent literature both the terms “IFN signature” and “type I IFN signature” are used to encompass the overexpression of genes induced by type I IFNs.^18,20^ Diseases in which this signature appears to play a prominent role are SLE, Sjögren’s syndrome, inflammatory myositis and scleroderma.^17,19,21–24^

Although extensive research has been conducted, there is still debate about various aspects regarding the measurement of the IFN signature. First of all, there is no universally accepted combination of genes to be analysed for the calculation of the IFN score, which is classically assessed by the expression level of different IFN- induced mRNA.^17,19^ In the most recent literature, four or five gene sets^19,21^ have been used when evaluating the IFN signature in autoimmune rheumatic diseases (ARDs). In addition, the transcriptomic overlap between distinct types of IFNs, is still a limitation,^23,25^ although the development of new techniques allows the differentiation of type I IFNs.^24,26^ The type of IFN producer cells to analyse has also shifted from the initial focus on plasmacytoid DCs to tissue-resident immune cells, keratinocytes, renal tubular cells, salivary gland epithelial cells and neutrophils.^25–29^ The causes or triggers of the IFN signature in ARDs are also not clear.^12^ Finally, the implications of IFN signature measurement in clinical practice needs to be explored, in order to determine its relevance for patient stratification and optimisation of ARD management.^17,19^

## IFNs IN SLE

SLE patients are characterised by increased levels of IFN in serum, a fact that has been known since the late 70s.^28,30^ Inherited mutations causing activation of the type I IFN pathway result in a lupus-like phenotypic activation of systemic autoimmunity.^29,31^ Expression level of IFN-induced genes correlates with SLE activity and severity, including active renal disease.^12^ As previously mentioned, although pDCs are probably the main source of IFN production in SLE, several other cell types contribute to the IFN signature, either by producing IFN themselves, or by stimulating pDC to an increased IFN production, contributing to the sustained autoimmune process in SLE.^12^ Circulating immune-complexes (ICs) seem to play a major role in the excessive activation of pDCs in SLE. This is supported by in vitro studies showing that DNA-containing ICs from active SLE patients’ serum, activate the innate immune system by inducing pDCs to produce IFN-α, and other pro-inflammatory cytokines and chemokines.^30,32^ Studies on healthy first-degree relatives of SLE patients have shown elevated serum IFN-α levels compared to healthy unrelated individuals.^31,33^ suggesting that an underlying genetic susceptibility is also required for producing high IFN-α levels in SLE.

IFNs have pleiotropic actions on various innate and adaptive immunity cells, namely activation and differentiation of B cells into plasma cells, increased T-cell proliferation and activation, impaired function of regulatory T-cells, and BAFF upregulation by dendritic cells, all of which contribute to SLE pathogenesis.^32,34^ This high IFN signature seems to have a major impact on the full range of clinical manifestations in SLE. Specifically, increased expression of IFN-regulated genes has been observed in epidermis and dermis of cutaneous lesions^33,35^ and the IFN signature has been demonstrated to correlate with cutaneous disease activity, suggesting a key role of IFN signalling in SLE skin pathology.^34,36^ However, the exact interplay between different IFNs, keratinocytes and pDCs needs further exploration**.** Increased expression of IFN-induced genes has been also demonstrated in synovial tissue from patients with SLE and inflammatory arthritis, probably deriving from fibroblasts, which are abundant in this tissue.^35,37^

The IFN-signature seems to also contribute to the severe SLE manifestations. Specifically, kidney biopsies of patients with lupus nephritis have shown increased expression of IFN-inducible genes, while pDCs accumulate in glomeruli of patients with active renal disease.^36,38^ Moreover, high IFN expression in peripheral blood correlates with LN severity.^37,39^ Studies on SLE patients with neuropsychiatric manifestations (NPSLE) have demonstrated that immune complexes formed by cerebrospinal fluid (CSF) autoantibodies are potent inducers of IFN-α, which is known to be increased in NPSLE patient’s CSF.^39–41^ In addition, IFN-α has been shown to activate microglia leading to synaptic pruning in lupus-prone mouse models and therapeutic administration of type I interferons induces psychiatric symptoms.^38,40^

## TARGETING THE IFN SYSTEM

It is now understood that IFNs play a critical role in the pathogenesis of SLE, which explains the numerous attempts to develop agents that inhibit the IFN pathway during the past decades. Multiple biologics have been developed targeting the type I IFN pathway, including monoclonal neutralising antibodies binding to IFN-α (sifalimumab, rontalizumab, JNJ-55920839 and AGS-009) or its receptor (anifrolumab), as well as a unique anti-IFN-α vaccine strategy (IFNα-kinoid).^40–43^

### Anifrolumab

Anifrolumab, previously known as MEDI-546, is the first biologic targeting the IFN system to be approved for SLE.^42,44^ It is a fully human, IgG1κ monoclonal antibody, able to bind to IFN-α/β receptor (IFNAR), leading to prevention of signal transmission by all type I IFNs.^42,44^ Recent phase II and III trials have proved its efficacy and safety in active SLE,^41–45^ summarised in **Table 1**.

**Table 1.** Completed trials on anifrolumab.

**Title**	**Main ID**	**Year onset completion**	**Trial type**	**Study population**	**Primary endpoint achievement**
Anifrolumab PK Study for Systemic Lupus Erythematosus (SLE)	NCT05001698	2021–2022	phase 1, open label, multiple dose	Chinese SLE patients, 18–60 years	NA
Anifrolumab Early Access Program (AMANA)	NCT04750057	2021 (no longer available)	open label, early access	SLE patients, moderate to severe active SLE	NA
A Study to Characterize the Pharmacokinetics, Pharmacodynamics, and Safety of Anifrolumab in Adult Type I Interferon Test High Systemic Lupus Erythematosus Subject With Active Skin Manifestations	NCT02962960	2016–2018	phase 2, placebo controlled, double blind	SLE patients, aged 18 – 70, CLASI≥ 10	Yes
Long Term Safety of Anifrolumab in Adult Subjects With Active Systemic Lupus Erythematosus TULIP SLE LTE	NCT02794285	2016–2021 TULIP SLE LTE	phase 3 extension, placebo controlled, double blind	SLE patients having completed one of TULIP trials	Yes
Safety and Efficacy of Two Doses of Anifrolumab Compared to Placebo in Adult Subjects With Active Proliferative Lupus Nephritis (TULIP-LN1)	NCT02547922	2015–2021 TULIP-LN	phase 2, placebo controlled, double blind	SLE patients, aged 18 – 70, Class III or Class IV LN	No
Efficacy and Safety of Two Doses of Anifrolumab Compared to Placebo in Adult Subjects With Active Systemic Lupus Erythematosus	NCT02446912	2015–2018 TULIP-I	phase 3, placebo controlled, double blind	SLE patients, aged 18–70, moderate to severe active SLE, 2 doses of anifrolumab	No
Efficacy and Safety of Anifrolumab Compared to Placebo in Adult Subjects With Active Systemic Lupus Erythematosus	NCT02446899	2015–2018 TULIP-2	phase 3, placebo controlled, double blind	SLE patients, aged 18–70, moderate to severe active SLE	Yes
An Open-label Study to Evaluate the Long-term Safety of MEDI-546, for the Treatment of SLE, in Adults	NCT01753193	2013–2018	phase 2, open label, extension	SLE patients, aged 18–68, moderate to severe SLE	Yes
Safety and Tolerability of Intravenous Dose of MEDI-546 in Japanese Subjects With Systemic Lupus Erythematosus	NCT01559090	2012–2017	phase 2, open label, dose escalation	SLE patients, aged 18–65, moderate to severe active SLE	Yes
A Study of the Efficacy and Safety of MEDI-546 in Systemic Lupus Erythematosus	NCT01438489	2012–2015 MUSE	phase2, placebo controlled, double blind	SLE patients, aged 18–65, moderately to severe SLE	Yes

CLASI; Cutaneous Lupus erythematosus Disease Area and Severity Index, LN; Lupus Nephritis, LTE; Long Term Extension Study, NA; Not Applicable, PK; Pharmacokinetics, SLE; Systemic Lupus Erythematosus

MUSE was a phase IIb, randomised, double-blind, placebo-controlled study,^42,44^ which evaluated anifrolumab’s efficacy and safety in adult SLE patients with moderate to severe disease activity, as an add-on to SOC. More patients on anifrolumab achieved a SLE Responder Index (SRI) 4 response^44,46^ at week 24 compared to placebo. Serious adverse event rates were similar across groups; however, herpes zoster and influenza incidence were more frequent on anifrolumab treated patients. A post hoc analysis of the MUSE study^45,47^ showed a greater response in rash and arthritis resolution in anifrolumab treated patients with high IFN signature, while an open label extension study showed sustained disease activity with comparable serious adverse events to those reported in the randomised controlled trial (RCT) phase.^46,48^

TULIP-1 was the first phase III RCT to be conducted on the use of anifrolumab in active SLE, whose primary endpoint was not met.^47,49^ However, several secondary endpoints were reached, which led to TULIP-2, the second phase III RCT on anifrolumab, with similar design,^43,45^ where a different primary endpoint, than in the TULIP-1 was used, the British Isles Lupus Assessment Group–Based Composite Lupus Assessment (BICLA) response.^48,50^ In this study, patients received placebo (n=182) or 300mg anifrolumab (n=180) every 4 weeks for a year’s period. The percentage of patients with a BICLA response was greater in the anifrolumab than in the placebo group (47.8% vs 31.5% respectively). Some secondary end points were also reached, including steroid dose reduction and Cutaneous Lupus Erythematosus Disease Area and Severity Index (CLASI)^49,51^ improvement. Herpes zoster, upper respiratory tract infection and bronchitis were more frequent in the anifrolumab arm.

Post-hoc analysis of both TULIP trials showed diminished overall disease activity combined with sustained glucocorticoid tapering in patients on anifrolumab compared to placebo^50,52^ and lower annualised flare rates, with longer periods of remission.^51,53^ A long-term extension of both TULIP trials (TULIP LTE) further assessed its safety for an additional 3 year period, showing no alterations in the safety profile of anifrolumab, with SAE, malignancy and major cardiovascular events being equivalent across the two arms.^52,54^

Following approval of anifrolumab for SLE patients with non-renal, active SLE, the first attempt to assess its efficacy in patients with active, biopsy proven, Class III/IV LN in the TULIP-LN phase II RCT^53,55^ did not achieve the desired results. Patients were assigned to receive either placebo or anifrolumab added to SOC treatment with the change in baseline 24-hour urine protein-creatinine ratio (UPCR) at week 52 as the primary endpoint. An extension study of TULIP-LN including patients who achieved at least partial renal response and steroid tapering target in the original RCT, supports further investigation of an anifrolumab intensified dosing regimen in patients with active proliferative LN.^54,56^ Safety profile illustrated a greater herpes zoster incidence with anifrolumab than placebo, while SAE were equivalent among groups.^53–56^ Finally, a phase II pharmacokinetics/pharmacodynamics (PK/PD), safety and efficacy, RCT evaluated the subcutaneous administration of anifrolumab in SLE patients with active skin disease and high Type I IFN levels, over SOC treatment.^55,57^ Overall, PK/PD, safety and efficacy profile, supported the further development of subcutaneous anifrolumab for SLE treatment.

Several ongoing trials on anifrolumab, summarised in **Table 2**, are expected to further characterise its profile. Importantly, anifrolumab on active proliferative nephritis will be evaluated in a phase III trial (IRIS, NCT05138133).^56,58^ An open label study from Japan will assess anifrolumab administration early in disease course, prior to other immunosuppressive or immunomodulatory drugs (jRCTs031230358),^57,59^ while further studies on subcutaneous anifrolumab administration will be conducted on moderate to severe SLE (TULIP SC NCT04877691),^58,60^ as well as on cutaneous lupus erythematosus refractory to first treatment line (LAVENDER, NCT06015737).^59,61^ Finally, a phase III PK/PD, efficacy and safety trial on anifrolumab administration in paediatric SLE is expected to start recruiting soon (NCT05835310).^60,62^

**Table 2.** Recruiting trials on anifrolumab.

**Title**	**Main ID**	**Year onset completion**	**Trial type**	**Study population**
Exploratory study for the usefulness of early introduction of anifrolumab in the first remission induction therapy for systemic lupus erythematosus	jRCTs031230358	2023-	single arm - open	SLE patients aged 18 – 80, within 6 months from diagnosis, not having received immunosuppressive or immunomodulatory drugs
A Study to Investigate the Efficacy and Safety of Anifrolumab in Adults With Chronic and/or Subacute Cutaneous Lupus Erythematosus (LAVENDER)	NCT06015737	2023–2026	phase 3, placebo controlled - double blind, followed by an open-label period	CLE patients, aged 18 – 70
Retrospective Medical Chart Review Study to Describe the Experience of SLE Patients Treated With Anifrolumab in the Early Access Programs ERYTHRO	NCT06046534	2023–2024	phase 3, retrospective observational	SLE patients ≥18years, ≥6 months on anifrolumab treatment
An Efficacy and Safety Study of Intravenous Anifrolumab to Treat Systemic Lupus Erythematosus in Pediatric Participants SLE	NCT05835310	2023–2029	phase 3, placebo controlled - double blind	SLE patients aged 5 - < 18
A Treatment Effectiveness Study Among SLE Patients Receiving Anifrolumab in Routine Clinical Practice ASTER	NCT05637112	2023–2029	prospective observational, treatment effectiveness	SLE patients ≥18years, initiating anifrolumab
The Role of Anifrolumab in Improving Markers of Vascular Risk in Patients With Systemic Lupus Erythematosus (SLE) IFN-CVD	NCT05440422	2023–2024	phase 2, placebo controlled, double blind, evaluating role of anifrolumab in modulating vascular function and inflammation	SLE patients, aged 18–80
SAPHNELO Systemic Lupus Erythematosus Japan Post-Marketing Surveillance (PMS)	NCT05141201	2021–2025	prospective observational	SLE patients, all ages, non responding to other therapy
Phase 3 Study of Anifrolumab in Adult Patients With Active Proliferative Lupus Nephritis (IRIS)	NCT05138133	2022–2028	phase 3, placebo controlled, double blind	patients with active proliferative LN Class III or IV
Anifrolumab Asian Phase III Efficacy Study for Systemic Lupus Erythematosus (SLE)	NCT04931563	2021–2025	phase 3, placebo controlled, double blind	Asian SLE patients, aged 18 – 70
Subcutaneous Anifrolumab in Adult Patients With Systemic Lupus Erythematosus Tulip SC	NCT04877691	2021–2025 TULIP-SC	phase 3, placebo controlled, double blind	SLE patients, aged 18 – 70, sc treatment
Nature of Anifrolumab Impact on Vaccine-Emergent Immunity in SLE (NAIVE)	NCT04726553	2021–2023	open label, impact on vaccine emergent immunity	SLE patients, aged 18–70, moderate to severe active SLE

CLE; Cutaneous Lupus erythematosus, CVD; Cardiovascular Disease, IFN; Interferon, LN; Lupus Nephritis, LTE; Long Term Extension Study, SC; Subcutaneous, SLE; Systemic Lupus Erythematosus.

### Anti-IFN monoclonal antibodies

Two monoclonal antibodies targeting specifically IFN-α, sifalimumab and rontalizumab, have been studied in phase I and II clinical trials. Sifalimumab is a human monoclonal antibody that directly targets IFN-α.^61,63^ Several RCTs^61–65^ and an open label study64,66 showed promising results regarding tolerability and safety of sifalimumab, while its primary endpoint was met in a phase 2 study, with a higher percentage of patients achieving SRI-4 in the treatment group.63,65 However, its development was discontinued in favour of anifrolumab, which showed more favourable results as described above.^41,43,63,65^ Rontalizumab, a humanised IgG1 monoclonal antibody, designed to neutralise all known IFN-α subtypes,^65,67^ demonstrated an acceptable safety profile in a phase I and a phase II study in SLE patients,^65–68^ while its efficacy was not proved as compared to placebo, leading to discontinuation of its development.

Another human monoclonal antibody targeting the majority of IFN-α subtypes, as well as IFN-ω, JNJ-55920839,^67,69^ was well tolerated in healthy adults and SLE patients with mild to moderate disease activity in phase I study,^67–70^ while an improvement in several disease indexes was recorded.^68,70^ There are no registered phase II studies for this agent yet. Finally, although AGS-009, a humanised anti-INFα monoclonal antibody neutralising various IFN-α subtypes, showed good safety profile in a phase Ia RCT in adults with mild to moderate SLE,^40,42^ it was not further developed.

### IFNα-kinoid

IFNα-kinoid (IFN-K) is a vaccine constructed as a therapeutic agent combining inactivated IFN-a2b with a T-helper carrier protein. Mathian et al. uncovered its potency to induce polyclonal antibodies neutralising all 13 subtypes of human IFN-α in human IFN-α transgenic mice, without affecting IFN-β or IFN-γ.^69,71^ Results from a multicentre, phase I/IIa staggered dose-escalation trial in adult SLE patients immunised with IFN-K, proved its efficacy on developing anti-IFNα antibodies. IFN signature-positive patients had both higher anti-IFNα titres and a reduced expression of IFN-induced genes. Higher anti-IFNα antibody titre was associated with IFN score decrease and C3 complement increase.^70,72^ An extension of this study further showed a diminished expression of genes involved in B cell activation following IFN-α neutralisation, and that antibody response induced by IFN-K had a polyclonal effect on 13 IFNα subtypes.^71,73^ Severity of adverse events in terms of injection site or systemic reactions was mild or moderate.^70,72^

**Figure 1.**
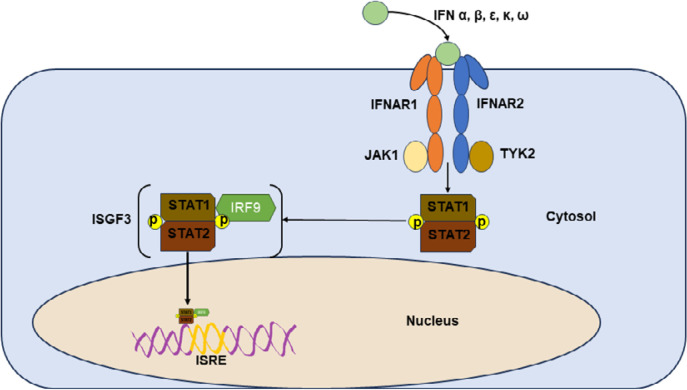
Induction mechanism of genes by type I interferons. After their release, Type I IFNs all bind to the same ubiquitously expressed type I IFN receptor (IFNAR) that consists of two polypeptide chains of IFNAR1 and IFNAR2. Subsequently, the signalling pathway involves activation of Janus kinase (JAK) 1 and tyrosine kinase (TYK) 2 and formation of the interferon-stimulated gene factor 3-complex (IGSF3), which includes signal transducer and activator of transcription (STAT)1, STAT2 and interferon regulatory factor (IRF). IGSF3 moves into the cell nucleus and binds to specific nucleotide sequences, called IFN-stimulated response elements (ISREs), which induce new gene transcription (IFN-regulated genes) in order to mediate antiviral responses.

Another phase IIb RCT, showed a reduction of IFN gene signature and a strong polyclonal immunogenic response in 91% of immunised patients. Despite the fail to meet a favourable change in response rate measured by BICLA, Lupus low disease activity state (LLDAS) was achieved in more patients administered IFN-K than placebo, also allowing more steroid reduction.^72,74^ Recorded adverse events were about the same among the two arms of the study, namely mild infections, headaches, nasopharyngitis and arthralgia and injection site induration.^72,74^

#### Indirect targets of the IFN system

Several other agents indirectly affect the IFN pathway, among which JAK/TYK inhibitors, immunomodulators that are successfully used for the treatment of several autoimmune diseases, such as rheumatoid arthritis.^75^ By inhibiting the activity of one or more of the JAK family of enzymes (JAK1, JAK2, JAK3, TYK2), they interfere with the JAK-STAT signalling pathway in lymphocytes and downregulate IFN signalling.^75^ In SLE patients, Deucravacitinib, a TYK 2 inhibitor and tofacitinib both managed to reduce type I interferon gene signature in early studies.^76,77^ Contrarily, several other trials testing JAK inhibitors in SLE patients failed to reach the pre-specified endpoints.^78,79^

Litifilimab (BIIB059) is a humanised IgG1 monoclonal antibody targeting blood dendritic cell antigen 2 (BDCA2) reducing, among other cytokines, plasmatocytoid dendritic cells’ type I IFN production, which has already proved efficacious in a phase II trial of CLE patients.^80^ Phase III studies, in SLE and CLE are awaited to further assess its efficacy.^81–84^

Dapirolizumab pegol (DZP) is a polyethylene glycol-conjugated antigen-binding fragment, targeting CD40L which, after its proved efficacy in active SLE,^85^ showed decreased expression of type I IFN signature in patients with high baseline type I IFN expression.^86^ Finally, GSK2646264, a spleen tyrosine kinase (SYK) inhibitor with topical application, showed a modest incline of several interferon-related genes, in patients with cutaneous lupus erythematosus (NCT02927457).^87^

## CONCLUSIONS

Our understanding of how the IFN system impacts on the sustained autoimmune process in SLE continues to progress. Nevertheless, there are still challenges to overcome to unlock the complexity of targeting the IFN pathway in a multifaceted disease like SLE. Further clinical trials on the use of anifrolumab in severe SLE complications and results from ongoing trials of novel IFN inhibitors are eagerly awaited.

## ABBREVIATIONS

ARD: Autoimmune Rheumatic Disease
BDCA2: Dendritic Cell Antigen 2
BICLA: British Isles Lupus Assessment Group–Based Composite Lupus Assessment
CLASI: Cutaneous Lupus Erythematosus Disease Area and Severity Index
CSF: cerebrospinal fluid
GASs: IFN-γ-activated sites
IFN: interferon
IFNAR1: type I interferon (IFN) receptor subunit 1
IFNGR: IFN-γ receptor
IGSF3: interferon-stimulated gene factor 3-complex
IL: interleukin
IRF: interferon regulatory factor
ISREs: IFN-stimulated response elements
JAK: Janus kinase
LLDAS: Lupus low disease activity state
LN: Lupus nephritis
MHC: major histocompatibility complex
NK: Natural Killer cells
NLRs: nucleotide oligomerisation domain–like receptors
NPSLE: Neuropsychiatric SLE
pDC: plasmacytoid dendritic cell
PK/PD: pharmacokinetics/pharmacodynamics
PRRs: pattern recognition receptors
RCT: Randomised controlled trial
RIG-I: retinoic acid inducible gene 1 (RIG-I)–like receptors
RLRs: RIG-I–like receptors
SAE: Serious adverse event
SLE: Systemic lupus erythematosus
SOC: Standard-of-care
SRI: SLE Responder Index
STAT: signal transducer and activator of transcription
TLRs: Toll-like receptors
TYK: tyrosine kinase
UPCR: urine protein-creatinine ratio
